# Annexin A4 induces platinum resistance in a chloride-and calcium-dependent manner

**DOI:** 10.18632/oncotarget.2306

**Published:** 2014-08-04

**Authors:** Akiko Morimoto, Satoshi Serada, Takayuki Enomoto, Ayako Kim, Shinya Matsuzaki, Tsuyoshi Takahashi, Yutaka Ueda, Kiyoshi Yoshino, Masami Fujita, Minoru Fujimoto, Tadashi Kimura, Tetsuji Naka

**Affiliations:** ^1^ Department of Obstetrics and Gynecology, Osaka University Graduate School of Medicine, Japan; ^2^ Laboratory for Immune Signals, National Institute of Biomedical Innovation, Japan; ^3^ Department of Obstetrics and Gynecology, Niigata University Medical School, Japan; ^4^ Department of Surgery, Osaka University Graduate School of Medicine, Japan

**Keywords:** Annexin A4, platinum resistance, annexin repeat, chloride ion

## Abstract

Platinum resistance has long been a major issue in the treatment of various cancers. We previously reported that enhanced annexin A4 (ANXA4) expression, a Ca^2+^-regulated phospholipid-binding protein, induces chemoresistance to platinum-based drugs. In this study, we investigated the role of annexin repeats, a conserved structure of all the annexin family, responsible for platinum-resistance as well as the effect of knockdown of ANXA4. ANXA4 knockdown increased sensitivity to platinum-based drugs both *in vitro* and *in vivo*. To identify the domain responsible for chemoresistance, ANXA4 deletion mutants were constructed by deleting annexin repeats one by one from the C terminus. Platinum resistance was induced both *in vitro* and *in vivo* in cells expressing either full-length ANXA4 or the deletion mutants, containing at least one intact annexin repeat. However, cells expressing the mutant without any calcium-binding sites in the annexin repeated sequence, which is essential for ANXA4 translocation from the cytosol to plasma membrane, failed to acquire platinum resistance. After cisplatin treatment, the intracellular chloride ion concentration, whose channel is partly regulated by ANXA4, significantly increased in the platinum-resistant cells. These findings indicate that the calcium-binding site in the annexin repeat induces chemoresistance to the platinum-based drug by elevating the intracellular chloride concentration.

## INTRODUCTION

Since cisplatin was first introduced as an anticancer drug in the 1970s [1], various platinum-based drugs have been developed and widely used not only against gynecological but also against other cancers, including lung, colorectal, testicular, prostate and bladder cancer [2-6]. Although these platinum-based drugs have significantly contributed to improve survival rates, chemoresistance to these drugs has become a major problem in recent years [7-9]. It has now been elucidated that the mechanism of platinum resistance is mediated by reduced platinum accumulation, increased platinum detoxification, increased repair of platinum–DNA adducts and inhibited apoptosis [10-12]. Several proteins have been reported to be candidate factors such as copper transporters: CTR1[13], ATP7A and ATP7B[14-17]; multidrug resistance protein 2 (MRP2) [18-20]; glutathione S-transferase enzyme π (GSTπ) [21]; excision cross-complementing gene 1 (ERCC1) [22]; receptor-interacting protein 1 (RIP1) [23]; microRNAs [24-26]; and p53 [27]. In contrast, there are still several proteins related to platinum resistance without a full understanding of how these proteins help cells to confer platinum-based drugs.

We recently reported that annexin A4 (ANXA4) is overexpressed in ovarian clear cell carcinoma and induces chemoresistance to platinum-based drugs [28]. Annexins are calcium-regulated and negatively charged phospholipid membrane-binding proteins. The basic structure of annexins consists of 2 major domains: a conserved structural element called an annexin repeat, a segment of 70 amino acid residues at the C terminus, and the N-terminal region unique for a given member of the family and determining individual annexin properties *in vivo*. The annexin repeat possesses the calcium and membrane binding sites and is responsible for mediating the canonical membrane binding properties [29]. These domains in ANXA4 are surrounded by relatively short amino and carboxy termini that do not have any known function [30]. ANXA4 is involved in membrane permeability, exocytosis and regulation of chloride channels in a calcium-dependent manner [29, 31-33]. ANXA4 is almost exclusively expressed in epithelial cells [34]. With regard to cancer, ANXA4 overexpression has been reported in various tumours, such as lung, gastric, colorectal, renal, pancreatic, ovarian and prostate cancer [28, 35-39] and is associated with tumour invasiveness, metastasis and chemoresistance [37, 40]. Moreover, ANXA4 has been shown to be associated with resistance to platinum-based drugs [28, 41-43].

ANXA4-induced platinum resistance appears to be mediated in part by the increased extracellular efflux of platinum mediated by the copper transporter ATP7A[28, 44]. Another mechanism of ANXA4-induced chemoresistance is the modulation of NF-κB transcriptional activity [45]. ANXA4 suppresses NF-κB transcriptional activity through interaction with the p50 subunit in a calcium-dependent manner; ANXA4 causes resistance to apoptosis induction by etoposide.

While ANXA4 prominently associated with chemoresistance, the functional domain of ANXA4 remains unclear. Therefore, to clarify the functional domain of ANXA4 is required to understand detailed mechanisms of the chemoresistance induced by ANXA4 and also overcome chemoresistance. In this study, focusing on platinum resistance, we aimed to identify the ANXA4 domain relevant to chemoresistance with regard to its structure as well as to test whether knockdown of ANXA4 expression could improve platinum resistance. Our data showed that the annexin repeat plays an important role in platinum resistance induced by ANXA4, which occurs in a calcium-dependent manner.

## RESULTS

### Establishment of ANXA4 knockdown RMG-I cells

**Fig.1:**
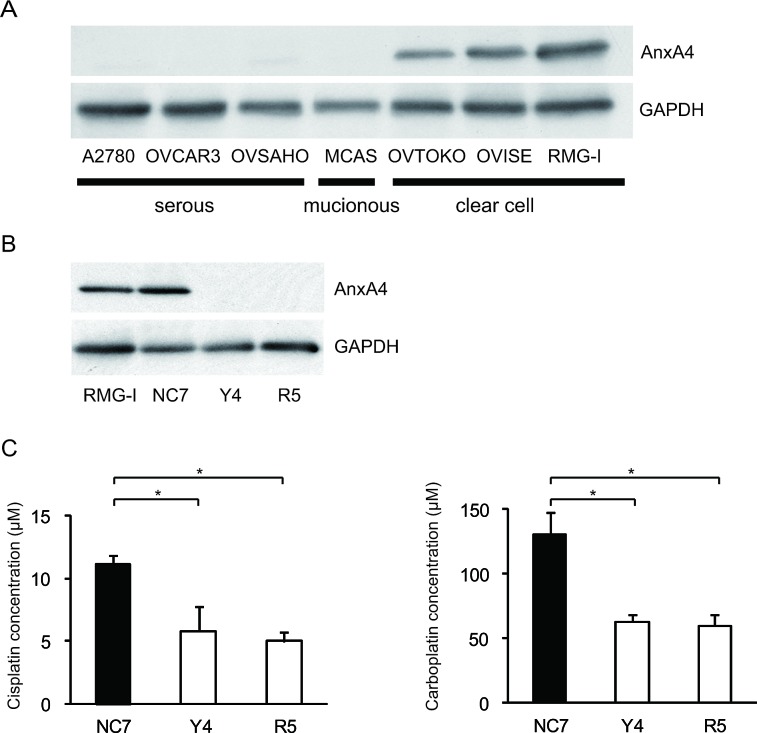
Knockdown of ANXA4 expression attenuates platinum resistance (A) ANXA4 expression in indicated ovarian cancer cell lines and (B) established ANXA4 knockdown RMG-I cells (R5 and Y4) was confirmed using Western blotting. (C) IC_50_ for both cisplatin and carboplatin was significantly reduced in R5 and Y4 cells compared with NC7 cells. Data are presented as mean ± SD (*p < 0.01).

To create cell lines with a stable ANXA4 knockdown, we analysed ANXA4 expression in ovarian cancer cells using western blotting. ANXA4 expression was strong in clear cell carcinoma cell lines (OVTOKO, OVISE and RMG-I) compared with serous adenocarcinoma cell lines (A2780, OVCAR3 and OVSAHO) and a mucinous adenocarcinoma cell line (MCAS; Fig. 1A). To see whether blocking ANXA4 expression was a valid chemosensitising strategy for ovarian clear cell carcinoma treatment, ANXA4 was stably suppressed using an ANXA4 shRNA plasmid. We established RMG-I-Y4 and R5 cell clones as well as RMG-I NC7 cell clones transfected with the empty vector as a control. Compared with RMG-I NC7 and untransfected control parent RMG-I cells, ANXA4 expression was markedly down-regulated at the protein level in RMG-I-Y4 and RMG-I-R5 cells (Fig. 1B). In the absence of any drug treatment, the growth rate among the 4 cell lines was similar *in vitro* (data not shown).

### Knockdown of ANXA4 expression enhances sensitivity to cisplatin and carboplatin

The sensitivity to cisplatin and carboplatin was assessed in the 3 RMG-I clones NC7, R5 and Y4. Compared with the IC_50_ for cisplatin in NC7 cells, IC_50_ was significantly decreased in Y4 cells and R5 cells (*p* < 0.01; Fig. 1C, left panel). Similarly, IC_50_ for carboplatin significantly decreased in Y4 cells and R5 cells compared with NC7 cells (*p* < 0.01; Fig. 1C, right panel). IC_50_ for cisplatin and carboplatin decreased approximately 2-fold because of the knockdown of ANXA4 expression.

### Suppression of ANXA4 expression improves platinum sensitivity *in vivo*

**Fig.2:**
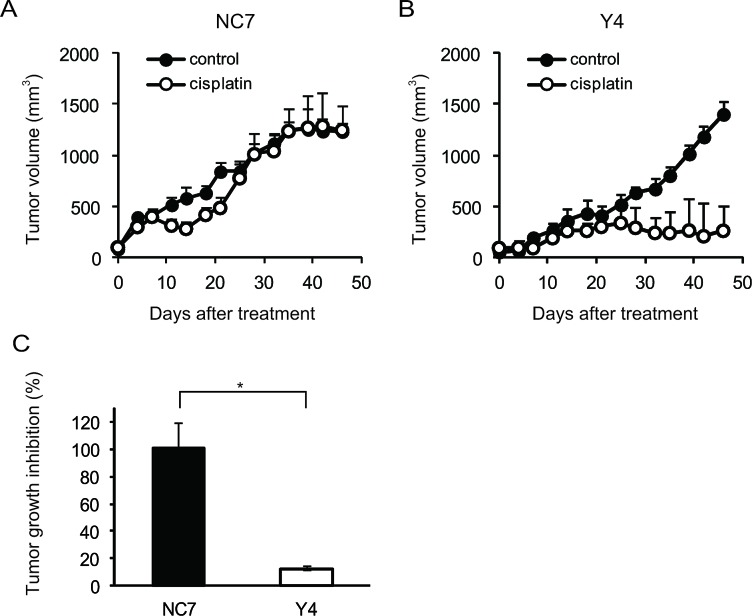
ANXA4 knockdown cells show enhanced sensitivity to cisplatin *in vivo* Female ICR *nu/nu* mice were subcutaneously inoculated with RMG-I NC7 or Y4 cells and given PBS (control group: *filled circles*) or cisplatin i.p. (3 mg/kg; treatment group: *open circles*) twice weekly for 4 weeks (n = 6 per group). Growth curves of NC7 tumours (A) and Y4 tumours (B). The mean volume (points) ± SE (bars) is shown. (C) Comparison of the cisplatin-induced growth inhibition of tumours 46 days after treatment among NC7 and Y4 tumours. The average (columns) ± SE (bars) are shown (*p < 0.01).

To determine whether ANXA4 knockdown in clear cell carcinoma cells improved platinum sensitivity *in vivo*, NC7 and Y4 cells were subcutaneously injected in ICR *nu/nu* mice. One week after inoculation with the tumour cells, the mice were randomised into 2 groups and received cisplatin or PBS *i.p.* twice a week for 4 weeks. The tumour growth rate in the absence of drugs was similar for both cell lines (Figs. 2A and 2B). Cisplatin treatment had very little effect on NC7 cells (Fig. 2A), but tumour volume markedly decreased in Y4 cells (Fig. 2B). Cisplatin treatment significantly decreased tumour growth in Y4 cells (87.4 ± 1.8%) compared with NC7 cells (−1.1 ± 18.0%; *p* < 0.01; Fig. 2C). These results showed that ANXA4 knockdown in the RMG-I cell line significantly attenuated resistance to cisplatin *in vivo*.

### The annexin repeat domain is required for the platinum drug resistance

**Fig.3:**
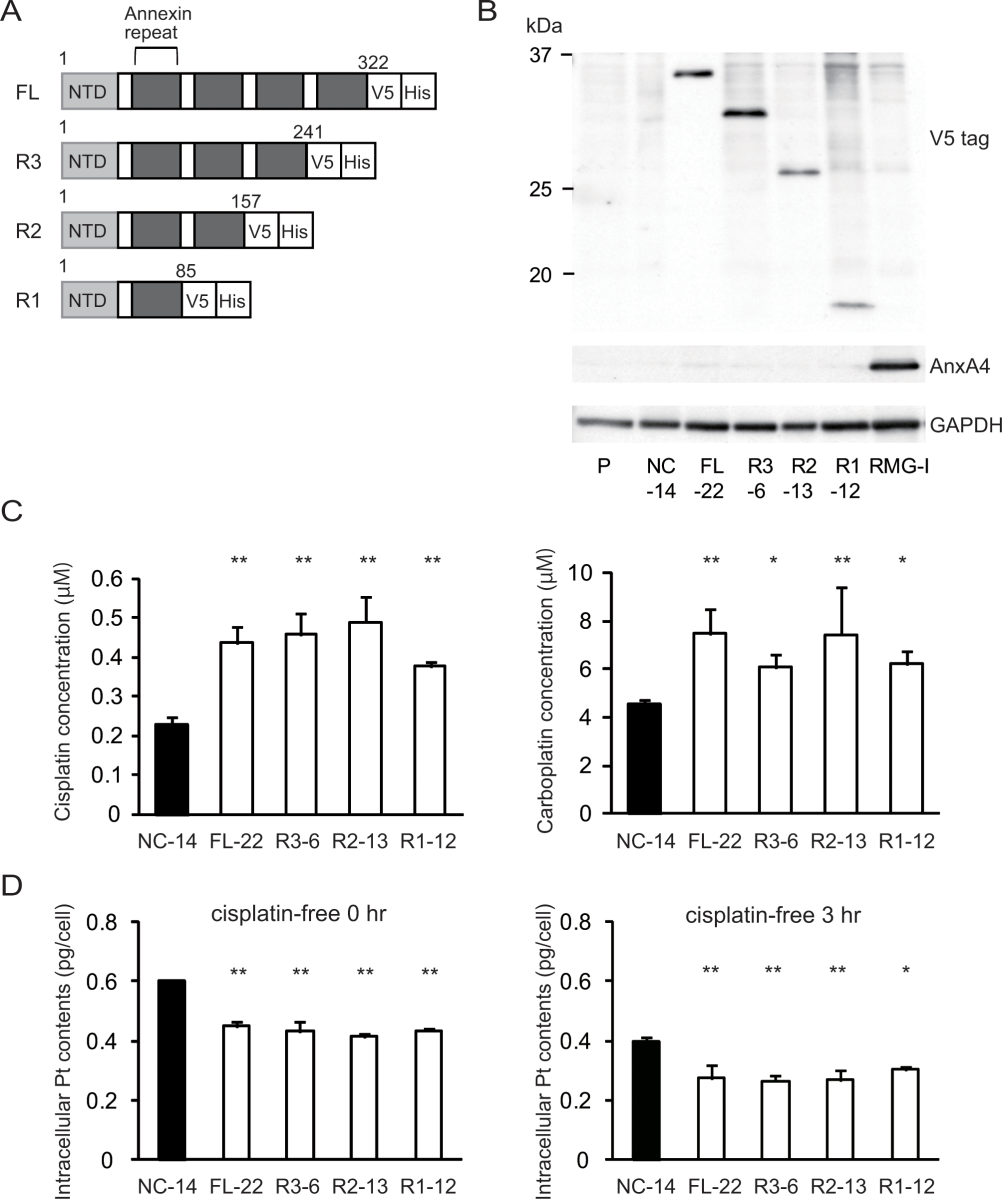
Annexin repeat domain is required for the platinum drug resistance (A) A structural map of ANXA4 and 3 deletion mutant proteins. Annexin repeats were deleted one by one from the C-terminal site. (B) Established deletion mutant cells together with parent cells, control cells and RMG-I as a positive control were confirmed using Western blotting. (C) Compared with NC-14 cells, IC_50_ for both cisplatin and carboplatin was significantly increased in FL-22 and all other mutant cells. (D) Intracellular platinum accumulation after treatment with 100 μM cisplatin for 60 min with or without additional 3 hr of incubation in cisplatin-free medium. Data are presented as mean ± SD (*p < 0.05, **p < 0.01).

To identify a possible resistance-related domain within the annexin repeated sequence of ANXA4, we constructed 3 deletion mutants by deleting the annexin repeats one by one from the C-terminal region. Figure 3A shows the structure of each deletion mutant. Full-length ANXA4, 3 ANXA4 deletion mutants or the empty vector were transfected into NUGC3 cells, whose endogenous ANXA4 expression is relatively low (Supplementary Fig. S1). Therefore, we established cell lines stably overexpressing full-length ANXA4 (FL-22), each ANXA4 deletion mutant (R3-6, R2-13 or R1-12) or the empty vector (NC-14). Expression of each ANXA4 deletion mutant was confirmed using Western blotting (Fig. 3B).

Subsequently, the sensitivity to the platinum-based drugs cisplatin and carboplatin was assessed. Cells transfected with full-length ANXA4 and the 3 deletion ANXA4 mutants were significantly more resistant to both cisplatin and carboplatin compared with control cells, approximately with a 1.7- to 2.2-fold increase in IC_50_ for cisplatin (*p* < 0.01) and a 1.4- to 1.7-fold increase in IC_50_ for carboplatin (*p* < 0.05; Fig. 3C).

To test whether these deletion mutants induce platinum resistance through regulating cellular drug concentration as previously reported [28], we quantitated the intracellular platinum content of each deletion mutant-transfected cell clone after cisplatin treatment, which is one of the most representative platinum drugs. Platinum accumulation was significantly reduced in cells overexpressing either full-length ANXA4 or any of the 3 deletion mutants compared with NC-14 cells regardless of the incubation time after cisplatin exposure (Fig. 3D). These results suggested that the decreased intracellular platinum contents were associated with platinum resistance of the cells transfected with ANXA4 full length and each deletion mutant.

### The calcium-binding site of the annexin repeat is responsible for platinum resistance

**Fig.4:**
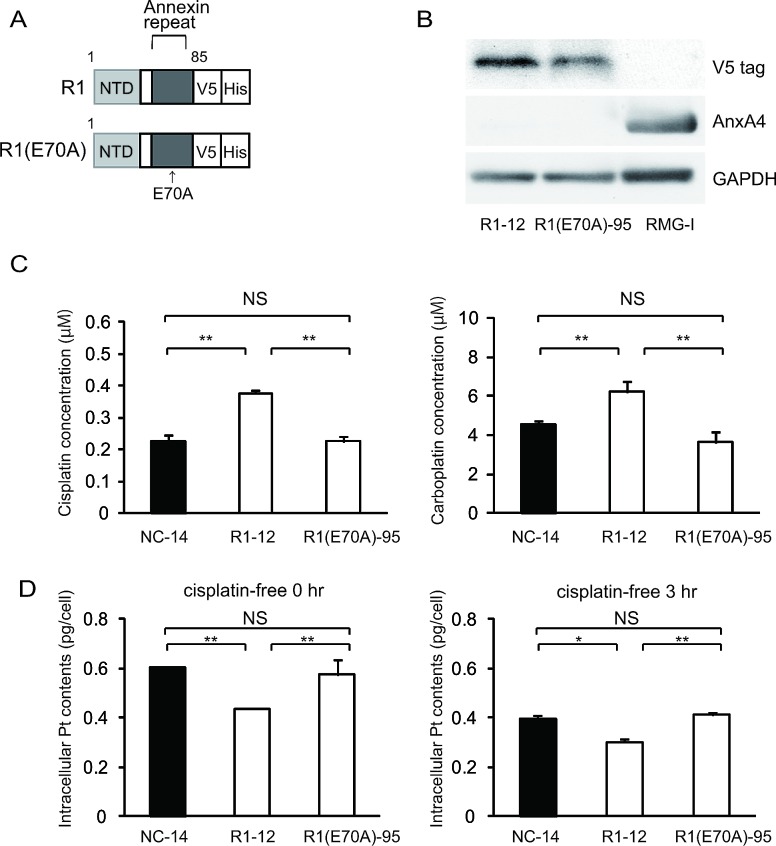
The calcium-binding site of the annexin repeat is responsible for induction of the platinum resistance (A) A structural map of R1 and R1(E70A) variants without a function for the calcium-binding site. (B) Western blotting confirmed the mutant established cell lines. (C) Unlike R1-12, the R1(E70A)-95 cell clone was not resistant to either cisplatin or carboplatin. (D) Intracellular platinum accumulation in R1(E70A) cells was the same as in NC14 cells both with or without additional 3 hr of incubation in cisplatin-free medium. Data are presented as mean ± SD (*p < 0.05, **p < 0.01).

As specified above, platinum resistance was enhanced in cells overexpressing ANXA4 deletion mutants, which contained at least 1 intact annexin repeat. Thus, to assess whether the calcium-binding site of the annexin repeat sequence was involved in chemoresistance, another deletion mutant, R1(E70A) was constructed. Within the annexin repeat next to the N-terminal region, the 70th amino acid, glutamic acid, was responsible for the calcium-dependent activity of ANXA4 [30]. Accordingly, at this site, the point mutation variant of R1, R1(E70A), loses the function of its calcium-binding site (Fig. 4A). Similar to other deletion mutants, R1(E70A) was transfected into NUGC3 cells and designated R1(E70A)-95. Western blotting revealed that R1-12 had the same molecular weight as R1(E70A)-95 (Fig. 4B). R1(E70A)-95 did not induce resistance to either cisplatin or carboplatin (Fig. 4C). Moreover, the intracellular platinum content of R1(E70A)-95-transfected cells did not decrease compared with that of NC-14 cells after 0 hr or 3 hr of additional incubation in cisplatin-free medium (Fig. 4D). According to the above results, the platinum resistance of ANXA4 seemed to be related to the calcium-binding site of the annexin repeat next to the N-terminal domain.

### The calcium-binding site of the annexin repeated sequence is required for the resistance to platinum-based drugs *in vivo*

**Fig.5:**
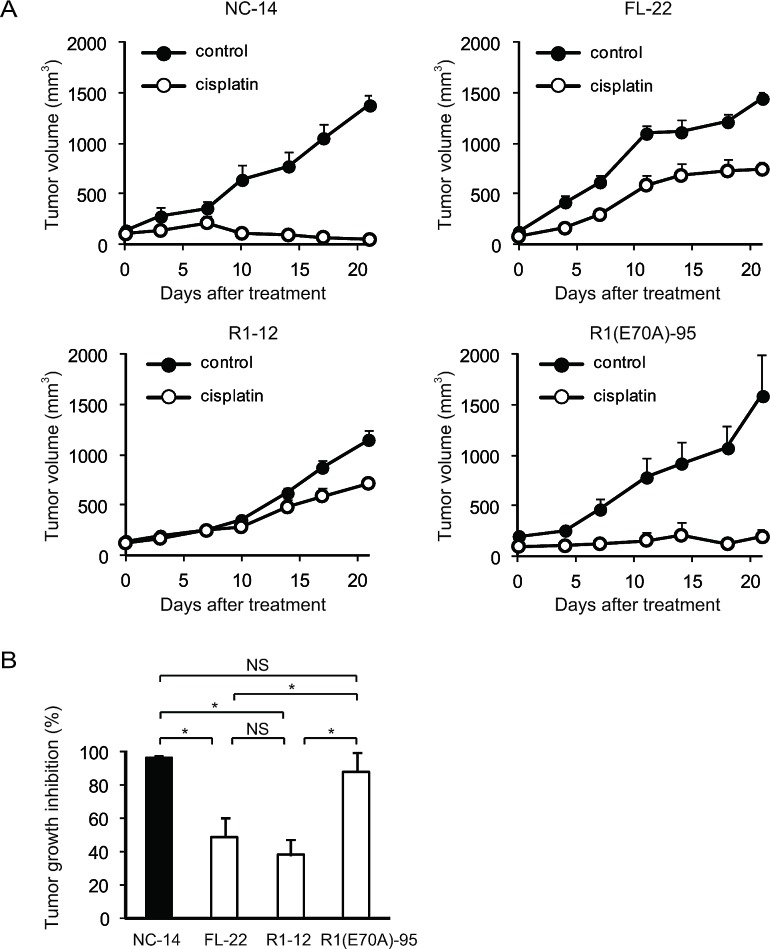
The calcium-binding site of the annexin repeat is required for platinum drug resistance *in vivo* Female ICR *nu/nu* mice were subcutaneously inoculated with NC14, FL-22, R1-12 or R1(E70A)-95 cells and given PBS (control group: *filled circles*) or cisplatin i.p. (3 mg/kg; treatment group: *open circles*) once a week for 3 weeks (n = 6 per group). (A) Growth curves of tumours of each cell. The mean volume (points) ± SE (bars) is shown. (B) Comparison of the cisplatin-induced growth inhibition of tumours 28 days after treatment. The average (columns) ± SE (bars) are shown; *p < 0.01.

To determine whether the annexin deletion mutants of ANXA4 influenced the sensitivity to cisplatin *in vivo*, we inoculated ICR *nu/nu* mice with NC-14, FL-22, R1-12 or R1(E70A)-95 cells. Mice in each group were randomised into 2 subgroups and received either cisplatin at 3 mg/(kg·d) or PBS *i.p.* once a week for 3 weeks. Cisplatin markedly decreased tumour volume in mice injected with NC-14 and R1(E70A)-95 cells, whereas the treatment effect was relatively smaller in mice injected with FL-22 and R1-12 cells (Fig. 5A). Consistent with the tumour volume, tumour growth was significantly inhibited by cisplatin in mice inoculated with NC-14 (96.5 ± 1.3%) and R1(E70A)-95 cells (87.8 ± 11.4%) compared with those injected with FL-22 (48.5 ± 11.7%) and R1-12 cells (37.7 ± 9.8%; *p* < 0.01; Fig. 5B). These *in vivo* results were consistent with those obtained *in vitro*.

### Increase of the intracellular chloride concentration is related to cisplatin resistance

To elucidate the molecular mechanisms of chemoresistance induced by ANXA4, we focused on the chloride channel because one of the functions of ANXA4 is inhibition of calcium-dependent chloride conductance[32]. According to the literature, treatment with cisplatin induces an increase of the intracellular Ca^2+^ concentration [46], which is an important ion for the phospholipid membrane-binding activity of ANXA4. In contrast, cisplatin exposure also induces an elevation of the intracellular chloride concentration: [Cl^−^]_i_ [47]. Elevation of [Cl^−^]_i_ has been shown to prevent the aquation of 1 or 2 of the 2 chloride coordination sites in cisplatin, and only the aquated forms of cisplatin covalently bind to DNA. Nevertheless, the mechanisms of [Cl^−^]_i_ elevation because of cisplatin treatment have not been fully elucidated. We hypothesised that an increase in intracellular Ca^2+^ concentration after cisplatin exposure would result in translocation of ANXA4 from the cytosol to plasma membrane, which leads to [Cl^−^]_i_ accumulation through inhibition of the chloride channel by the Ca^2+^-bound ANXA4. To confirm this hypothesis, we quantified [Cl^−^]_i_ after cisplatin treatment using MAQE fluorescence, a fluorescent Cl^−^ indicator. Relative fluorescence was substituted for [Cl^−^]_i_ as previously reported [48].

**Fig.6:**
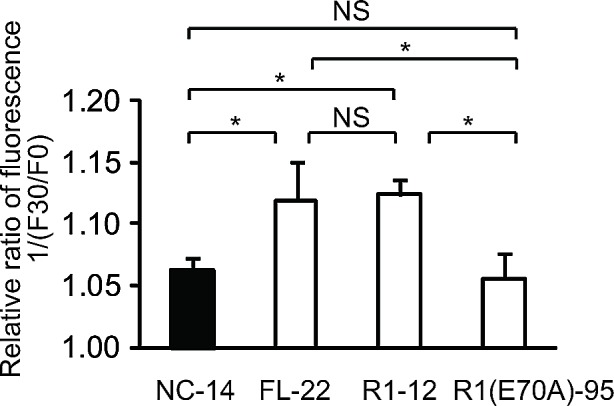
The increase of intracellular chloride concentration is related to cisplatin resistance ANXA4 deletion mutant series cells (NC-14, FL-22, R1-12 and R1[E70A]-95) loaded with N-ethroxycarbonymethyl-6-methoxyquinolinium bromide (MQAE) were exposed to 100 μM cisplatin. The fluorescence pre-treatment and during treatment (30 min exposure) was compared in each cell clone. Data are presented as mean ± SD (*p < 0.01).

We monitored MQAE fluorescence in control cells (NC-14), in cells overexpressing full-length ANXA4 (FL-22) and in 2 deletion mutants (R1-12 and R1[E70A]-95). The relative fluorescence ratio before (F0) and after treatment with cisplatin for 30 min (F30) is shown in Figure 6. The inverse ratio of MQAE fluorescence 1/(F30/F0), which is directly proportional to the increase in [Cl^−^]_i_, was significantly elevated in the platinum-resistant cell clones FL-22 (1.12 ± 0.03) and R1-12 (1.12 ± 0.01) compared with sensitive clones, NC-14 (1.06 ± 0.01) and R1(E70A)-95 (1.06 ± 0.02; *p* < 0.01). Thus, the increase in [Cl^−^]_i_ is likely to be involved in cisplatin resistance.

## DISCUSSION

ANXA4 has been reported to be strongly expressed and involved in chemoresistance in various cancers. The factors associated with ANXA4-induced chemoresistance have been reported, such as NF-κB [45] and ATP7A [44], but the structure of the protein, i.e. annexin repeats and calcium-binding sites in the annexin repeated sequence, has not been taken into account in relation to the ANXA4-induced chemoresistance. In this study, we showed that ANXA4 knockdown improved sensitivity to platinum drugs, and annexin repeats were involved in chemoresistance.

We first confirmed ANXA4 expression in various ovarian adenocarcinoma cell lines. As previously reported [28, 49], ANXA4 is significantly upregulated in clear cell carcinoma cell lines (OVTOKO, OVISE and RMG-I) compared with other histological types (serous and mucinous adenocarcinoma cell lines: A2780, OVCAR3, OVSAHO and MCAS). It has been previously demonstrated that enhanced ANXA4 expression induces platinum resistance both *in vitro* and *in vivo* [28, 44], but whether ANXA4 knockdown attenuates platinum resistance has been unknown thus far. Mogami et al. recently reported that an ANXA4 knockdown improves sensitivity to carboplatin *in vitro* using 2 cell lines of ovarian clear cell carcinoma, OVTOKO and OVISE. To the best of our knowledge, ours is the first study to show that ANXA4 knockdown markedly improves the sensitivity to platinum-based drugs not only *in vitro* but also *in vivo* (Figs. 1 and 2).

The result that ANXA4 knockdown improves sensitivity to platinum-based drugs suggests that ANXA4 is a good therapeutic target. To identify the functional domain(s) of ANXA4 that could be a promising therapeutic target, we focused on annexin repeats and constructed 4 deletion mutants (R3, R2, R1 and R1[E70A]). Resistance to platinum drugs was enhanced in cells transfected with mutants possessing at least 1 intact annexin repeat. In contrast, the sensitivity to platinum-based drugs improved among the R1(E70A)-transfected clones because in those cells, the calcium-binding site did not function properly (Figs. 3C and 4C). This result implies that the ANXA4-induced chemoresistance to platinum-based agents is calcium dependent. It has been reported that cisplatin induced increase of intracellular calcium concentration in chemosensitive cells, but not in resistant cells [46, 50]. Together with this and the data by Chan et al., elevation of intracellular calcium concentration induced by cisplatin treatment may translocate Ca^2+^ bound form of ANXA4 from cytosol to plasma membrane, which results in platinum-resistance[32]. We are currently investigating on further analysis.

By analysing the intracellular platinum accumulation, we attempted to elucidate the mechanism of the platinum resistance induced by ANXA4 and its deletion mutants. When intracellular platinum contents were quantitated just after exposure to cisplatin or 3 hr incubation with cisplatin-free medium after exposure to cisplatin, significantly less platinum accumulated in cells transfected with the full-length ANXA4 (FL-22) and 3 deletion mutants (R3-6, R2-13 and R1-12), all of which enhanced the resistance to the platinum-based drugs. In contrast, R1(E70A)-transfected cells (R1[E70A]-95), which did not induce chemoresistance, had the same amount of platinum accumulation as the control cells (Figs. 3D and 4D). These results suggest that the resistance to the platinum-based drugs is mediated by the decrease in intracellular platinum accumulation, which is calcium dependent. The annexin repeats, especially their calcium-binding sites, may be involved in inhibition of the influx, promotion of the efflux or both of platinum drugs. Recently, Cu transporters (CTR1 for the uptake and ATP7A and ATP7B for the efflux) have been reported to be involved in resistance to both cisplatin and carboplatin [14, 44, 51]. In addition, ANXA4 likely enhance platinum efflux through the interaction with ATP7A [44]. The possible mechanisms of inhibition of the influx mediated by ANXA4 remains unclear and further analyses are needed.

Subsequently, the question regarding the involvement of calcium-binding site in the platinum resistance arose. To answer this question, we measured [Cl^−^]_i_ after cisplatin exposure. The significant increase in [Cl^−^]_i_ was observed in the cells with platinum resistance, FL-22 and R1-12, compared with cell clones without platinum resistance, NC-14 and R1(E70A)-95. In a previous study, higher [Cl^−^]_i_ was observed in cisplatin-resistant cells compared with sensitive cells, whereas the intracellular cisplatin accumulation showed the opposite pattern [47]. These results, in addition to the results of decreased platinum accumulation in resistant mutants, indicate that ANXA4 induces platinum resistance through cellular drug efflux partly by elevating the intracellular chloride concentration. We report ‘partly’ because only cisplatin, not carboplatin, was tested in our [Cl^−^]_i_ assay. Cisplatin becomes intracellularly activated by the aquation of 1 or 2 of the 2 chloride coordination sites, but carboplatin does not contain any chloride coordination sites [1, 52-54]. Thus, this mechanism of resistance through elevation of [Cl^−^]_i_ may be specific to cisplatin and may not be true of carboplatin resistance. In this study, 3 cell clones overexpressing a deletion mutant (R3-6, R2-13, and R1-12) show stronger tolerance to cisplatin than to carboplatin in terms of their IC_50_; a 1.7- to 2.2-fold increase for cisplatin and only a 1.4- to 1.7-fold increase for carboplatin (Fig. 3C). It is assumed that the increase in [Cl^−^]_i_ is one of the factors inducing cisplatin resistance.

In this study, the calcium-binding site in the annexin repeat next to the N terminus was observed to be responsible for the resistance to the platinum drugs. Nevertheless, the role of the other 3 calcium-binding sites has not yet been investigated. The roles of individual calcium-binding sites were demonstrated using site-directed mutagenesis by Nelson and Creutz regarding calcium-dependent membrane binding and aggregation [30]. The mutations in each domain had different effects on the binding or aggregating activities, i.e. a mutation in the first or fourth domain had a greater effect on membrane binding. A mutation in the second domain had a stronger effect on membrane aggregation, whereas the mutation of the third domain was almost silent. Although the mechanisms involved in membrane binding/aggregation and the mechanisms of chemoresistance are likely different, our data could provide some clues to understanding the function of each annexin repeat and each calcium-binding site in chemoresistance.

ANXA4 has been shown to induce resistance to paclitaxel and platinum-based drugs [55]. The effect of ANXA4 knockdown on paclitaxel sensitivity was assessed in a previous study. The effect of sensitivity to paclitaxel varied among different cell clones: ANXA4 knockdown in the OVTOKO cell line with acidic isoelectric point (IEPs) did not improve the sensitivity to paclitaxel, whereas OVISE cell lines with basic IEPs showed improved sensitivity to paclitaxel [43]. In our own preliminary data, significant chemosensitisation to paclitaxel and etoposide was confirmed in RMG-I Y4 and R5 (data not shown). Further studies are required to identify the detailed mechanism.

In summary, in this study, we observed the annexin repeat, especially its calcium binding site, was associated with platinum-resistance induced by ANXA4, and it happened in calcium-dependent manner. Our findings may help to understand the mechanisms of platinum resistance induced by other annexin family proteins, which possesses the same annexin repeat structure, and offer new strategies for the treatment of chemoresistant cancers.

## METHODS

### Cell lines and culture

The human ovarian serous adenocarcinoma cell line (OVSAHO), human ovarian mucinous adenocarcinoma cell line (MCAS), human ovarian clear cell carcinoma cell lines (OVTOKO, OVISE and RMG-I) and human gastric cancer cell line (NUGC3) were obtained from the Japanese Collection of Research Bioresources (Osaka, Japan). A2780 cells from the human ovarian serous adenocarcinoma were obtained from the European Collection of Animal Cell Culture (Salisbury, Scotland) and OVCAR-3 cells from another human ovarian serous adenocarcinoma were from American Type Culture Collection (Manassas, VA). MCAS cells were maintained in the DMEM medium and the others in the RPMI medium, all supplemented with 10% foetal bovine serum (FBS; Serum Source International, NC, USA) and 1% penicillin–streptomycin (Nacalai Tesque, Kyoto, Japan) at 37°C in a humidified atmosphere with 5% CO_2_. All the cell lines were tested and authenticated.

### Generation of ANXA4 knockdown cell lines

To generate stable ANXA4 knockdown cell lines, RMG-I cells were transfected with a commercial plasmid vector expressing short heparin RNA (shRNA) that targeted ANXA4 mRNA or a negative control nonspecific shRNA (SuperArray Bioscience Corp., KH06928N; Frederick, MD, USA) using Lipofectamine 2000 (Invitrogen, Carlsbad, CA), according to the manufacturer's instructions. After selection using a culture medium containing geneticin (600 μg/mL; Invitrogen), stable clones were maintained in 250 μg/mL geneticin. Two stable RMG-I-ANXA4 shRNA cell clones were established, designated Y4 and R5 cells. In addition, we transfected the empty vector into the RMG-I cell line using the same procedure to generate control cells, designated NC7.

### Construction of ANXA4 deletion mutants and gene transfection

Using pcDNA3.1-ANXA4 as a template, full-length cDNA of ANXA4 was amplified using KOD-plus (Toyobo Co. Led., Osaka, Japan) with the primers forward 5′-TTGACCTAGAGTCATGGCCA-3′, reverse 5′-ATCATCTCCTCCACAGAGAA-3′ and subsequently ligated into the pcDNA3.1/V5-His-TOPO vector in-frame with the C-terminal V5 and 6× His tag. To generate ANXA4 deletion mutants, annexin repeat domains were deleted one by one from the C-terminal site. Three deletion mutants named R3, R2 and R1 were generated and similarly amplified (an Arabic number shows the number of annexin repeat domains). The nucleotide sequences of the forward primers for PCR were the same as those described above for all the deletion mutants, and the reverse primers were as follows: R3 5′-TATAGCCAGCAGAGCATCTT-3′, R2 5-CAGAGACACCAGCACTCGCT-3′ and R1 5′-CATCCCCACAATCACCTGCT-3′. We subsequently set out to generate an R1 mutant (the E70A mutation), whose calcium-binding site does not work because of the change of a negatively charged carboxyl group to a neutral side chain, as previously described [30]. The site-directed mutagenesis was performed using the KOD-Plus-Mutagenesis kit (Toyobo), according to the manufacturer's protocol. These cDNA fragments, including full-length gene, were subsequently inserted between the *Bgl* II and *EcoR* I sites of the pIRES2AcGFP vector (Clontech, Palo Alto, CA). The sequences of all the mutants were confirmed using the ABI PRISM 3100 Genetic Analyser (Applied Biosystems, Foster City, USA).

Full-length ANXA4, each ANXA4 deletion mutant construct and the empty vector were transfected into NUGC3 cells using Lipofectamine 2000 (Invitrogen). Stable transfectants designated FL-12, R3-6, R2-13, R1-12, R1(R70A)-95 and NC-14 were obtained by selection in a medium containing geneticin and were maintained in the same manner described above.

### Western blotting

Cells were lysed in RIPA buffer (10 mM Tris–HCl, pH 7.5, 150 mM NaCl, 1% Nonidet P-40, 0.1% sodium deoxycholate, 0.1% SDS, 1× phosphatase inhibitor cocktail (Nacalai Tesque) and 1× protease inhibitor cocktail (Nacalai Tesque)), followed by centrifugation (13,200 rpm, 4°C, 15 min). Soluble proteins in the supernatant were separated using sodium dodecyl sulphate polyacrylamide gel electrophoresis, as described previously [28]. Additional information can be found in the Supporting Information on Material and Methods section.

### Measurement of IC_50_ values after treatment with a platinum-based drug

Cells were suspended in the RPMI medium supplemented with 10% FBS, seeded in 96-well plates (1,500/well for the RMG-I series and 2,500 cells/well for ANXA4 deletion mutant series), cultured for 24 h and exposed to various concentrations of cisplatin (0–25 μM; Sigma–Aldrich, St Louis, MO) or carboplatin (0–1000 μM; Sigma–Aldrich) for 72 h. Cellular proliferation was subsequently evaluated using the WST-8 assay, i.e. 2-(2-methyosy-4-nitro-phenyl)-3-(4-nitrophenyl)-5-(2,4-disulfophynel)-2H-tetrazolium monosodium salt assay (Cell Counting Kit-SF; Nacalai Tesque) after treatment. The absorption of WST-8 was measured at a wavelength of 450 nm (reference wavelength: 630 nm) using a Model 680 microplate reader (Bio-Rad Laboratories, Hercules, CA). Absorbance values for the treated samples were expressed as percentages relative to results for untreated controls, and IC_50_ values were calculated.

### Measurement of intracellular platinum accumulation

Full-length ANXA4-transfected cells (FL-22), each ANXA4 deletion mutant-transfected cell clone (R3-6, R2-13, R1-12 and R1[E70A]-95), and control cells (NC-14) were cultured up to 80% confluence in 150-mm tissue culture dishes. The cells were then exposed to 100 μM cisplatin for 60 min at 37°C and washed twice with PBS either immediately or after 3 hr of incubation in cisplatin-free RPMI 1640 medium supplemented with 10% FBS. Whole-cell extracts were prepared, and the concentration of intracellular platinum was determined using an Agilent 7500ce inductively coupled plasma mass spectrometer (ICP-MS; Agilent, Santa Clara, CA, USA).

### *In vivo* model of cisplatin resistance

All animal experiments were conducted in accordance with the Institutional Ethical Guidelines for Animal Experimentation of the National Institute of Biomedical Innovation (Osaka, Japan). Female Institute of Cancer Research (ICR) *nu/nu* mice were obtained from Charles River Japan (Yokohama, Japan). Injection of the ANXA4 knockdown cells was performed as follows: ICR *nu/nu* mice at 4 weeks of age were subcutaneously inoculated (into the flank of the mice; n = 6 per group) with 2.5 × 10^6^ cells of RMG-I NC7 cells or RMG-I-Y4 cells in the total volume of 100 μL of 1/1 (v/v) PBS/Matrigel (Becton Dickinson, Bedford, MA). Injection of ANXA4 mutant-transfected cells, i.e. mice at 5 weeks of age were inoculated with 10^6^ cells of NC-14, FL-22, R1-12 or R1(E70A) in the same manner as with the ANXA4 knockdown cells. Treatment with cisplatin (3 mg/kg) or PBS *i.p.* was initiated 1 week after inoculation and administered twice weekly for 4 weeks (ANXA4 knockdown cells) and once a week for 3 weeks (ANXA4 mutant-transfected cells). Tumour volumes were determined twice weekly by measuring length (L), width (W) and depth (D) and using the following formula: tumour volume (mm^3^) = W × L × D.

### [Cl^−^]_i_ measurements

[Cl^−^]_i_ was measured using the fluorescent Cl^−^ indicator N-ethroxycarbonymethyl-6-methoxyquinolinium bromide (MQAE; Dojindo, Kumamoto, Japan). [Cl^−^]_i_ is detected by the mechanism of diffusion-limited collisional quenching of MQAE fluorescence. MQAE fluorescence intensity inversely correlates with [Cl^−^]_i_. The cells of the ANXA4 deletion mutant series (NC-14, FL-22, R1-12 and R1[E70A]-95) were cultured in 35-mm tissue culture dishes up to 20% confluence and incubated with a medium containing 10 mM MQAE for 4 h at 37°C. After loading, the cells were washed 5 times with Cl^−^-free buffer and electrically stimulated under a microscope at 37°C in a humidified atmosphere with 5% CO_2_. Fluorescence measurements were initiated immediately at the indicated periods using Biozero BZ-9000 (Keyence, Tokyo, Japan) at 510/40 nm excitation and 380/50 nm emission. The fluorescence was quantitated by means of a standardised procedure using a BZ-II Analyser (Keyence), and the data were presented as the reciprocal of the ratio of fluorescence data (F0/F30) to identify possible correlations with the increase in [Cl^−^]_i_.

### Statistical analysis

All calculations involved one-way analysis of variance (ANOVA) followed by Dunnett's analysis to evaluate the significance of differences. In all experiments, p value of <0.05 was considered statistically significant.

## SUPPLEMENTARY MATERIAL FIGURE


