# Supplementary material for: Annexin A4 induces platinum resistance in a chloride-and calcium-dependent manner
Source: Oncotarget. 2014 Aug 4;5(17):7776–87. doi: 10.18632/oncotarget.2306 (PMC4202160; doi:10.18632/oncotarget.2306)
Supplement: Supplementary file 1 [file oncotarget-05-7776-s001.pdf]

Annexin A4 induces platinum resistance in a chloride-and calcium-dependent manner

Supplementary Material

Supporting Information Materials and Methods

Cell lines and culture

The human hepatocellular cancer cell lines (HuH7), human prostate cancer cell line (LNCaP and DU145) were obtained from the Japanese Collection of Research Bioresources. LoVo, Caco2 and WiDr cells from the human colon cancer were obtained from American Type Culture Collection, Hep3B cells from the human hepatocellular cancer was from the Cell Resource Center for Biomedical Research (Tohoku University, Sendai, Japan) and PC3 cells from the human prostate cancer was from RIKEN BioResource Center Cell Bank (Tsukuba, Japan). Hep3B, HuH7, LoVo, Caco2 and WiDr cells were maintained in the DMEM medium and the others in the RPMI 1640 medium, all supplemented with 10% FBS.

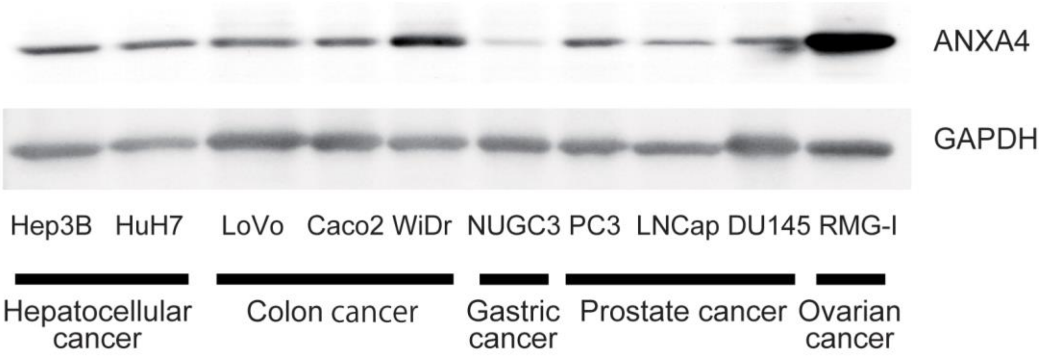

Figure S1: ANXA4 expression in cancer cell lines. ANXA4 expression was confirmed by Western blotting using 2 cell lines derived from hepatocellular cancer (Hep3B and HuH7), 3 from colon cancer (LoVo, Caco2 and WiDr), 1 from gastric cancer (NUGC3), 3 from prostate cancer (PC3, LNCap and DU145) and 1 from ovarian clear cell carcinoma (RMG-I) as a positive control.
